# Functional and Molecular Surveillance of *Helicobacter pylori* Antibiotic Resistance in Kuala Lumpur

**DOI:** 10.1371/journal.pone.0101481

**Published:** 2014-07-08

**Authors:** Xinsheng Teh, Yalda Khosravi, Woon Ching Lee, Alex Hwong Ruey Leow, Mun Fai Loke, Jamuna Vadivelu, Khean Lee Goh

**Affiliations:** 1 Department of Medical Microbiology, University of Malaya, Faculty of Medicine, Kuala Lumpur, Malaysia; 2 Department of Medicine, University of Malaya, Faculty of Medicine, Kuala Lumpur, Malaysia; The University of Hong Kong, Hong Kong

## Abstract

**Background:**

*Helicobacter pylori* is the etiological agent for diseases ranging from chronic gastritis and peptic ulcer disease to gastric adenocarcinoma and primary gastric B-cell lymphoma. Emergence of resistance to antibiotics possesses a challenge to the effort to eradicate *H. pylori* using conventional antibiotic-based therapies. The molecular mechanisms that contribute to the resistance of these strains have yet to be identified and are important for understanding the evolutional pattern and selective pressure imposed by the environment.

**Methods and Findings:**

*H. pylori* was isolated from 102 patients diagnosed with gastrointestinal diseases, who underwent endoscopy at University Malaya Medical Centre (UMMC). The isolates were tested for their susceptibility on eleven antibiotics using Etest. Based on susceptibility test, 32.3% of the isolates were found to have primary metronidazole resistance; followed by clarithromycin (6.8%) and fluoroquinolones (6.8%). To further investigate the resistant strains, mutational patterns of gene *rdxA*, *frxA*, *gyrA, gyrB*, and *23S rRNA* were studied. Consistent with the previous reports, metronidazole resistance was prevalent in the local population. However, clarithromycin, fluoroquinolone and multi-drug resistance were shown to be emerging. Molecular patterns correlated well with phenotypic data. Interestingly, multi-drug resistant (MDR) strains were found to be associated with higher minimum inhibitory concentration (MIC) than their single-drug resistant (SDR) counterparts. Most importantly, clarithromycin-resistant strains were suggested to have a higher incidence for developing multi-drug resistance.

**Conclusion:**

Data from this study highlighted the urgency to monitor closely the prevalence of antibiotic resistance in the Malaysian population; especially that of clarithromycin and multi-drug resistance. Further study is needed to understand the molecular association between clarithromycin resistance and multi-drug resistance in *H. pylori*. The report serves a reminder that a strict antibiotic usage policy is needed in Malaysia and other developing countries (especially those where *H. pylori* prevalence remained high).

## Introduction

*Helicobacter pylori* is an etiological factor for several gastro-duodenal diseases with various clinical manifestation ranging from dyspepsia, chronic gastritis, gastric atrophy to peptic ulcer disease [1]. In more severe cases, *H. pylori* infection may result in distal gastric adenocarcinoma and gastric mucosal-associated lymphoid tissue (MALT) lymphoma [2]. Therefore, it has been classified as the only known class I bacterial carcinogen by the International Agency for Research on Cancer (IARC) [3]. *H. pylori* is known to colonize almost half of the population worldwide with varying prevalence rates among different geographical regions with higher rates in developing countries [4]. In many developed societies the prevalence of infection rate has been noted to decrease from the late 90's onwards [5].

Early eradication of *H. pylori* has been proven to reduce the incidence rate of gastric cancer and improve ulcer healing [6], [7]. However, eradication of the pathogen has been difficult due to the limited antibiotics available that can withstand the low pH environment in stomach, or the capability of the drug to penetrate the gastric mucous layers to be in contact with the pathogen. To date, the most widely used treatment regimes include the standard triple therapy, which comprises two of three antibiotics, clarithromycin and amoxicillin or metronidazole, together with one proton pump inhibitor (PPI) [8]; and sequential therapy, which initiates treatment with proton pump inhibitor and amoxicillin followed by subsequent treatment with clarithromycin and metronidazole [9]. However, increasing prevalence of clarithromycin resistance greatly reduced the efficacy of those two therapies mentioned [10], [11]. As a counter measure, fluoroquinolone-containing triple and bismuth-containing quadruple therapies have also been proposed as second line therapies after the failure of the clarithromycin-containing treatment [12], [13]. In spite of that, a number of studies have shown a low but rapid rise of fluoroquinolone-resistance in countries where fluoroquinolone has been widely prescribed [14], [15], [16], [17], [18], [19]. To date, the mechanisms underlying the resistance to metronidazole, clarithromycin and flouroquinolones remains obscure.

At the University Malaya Medical Centre (UMMC), the first line eradication regimen for *H. pylori* consists of two antibiotics (amoxicillin 1 g twice daily and clarithromycin 500 mg twice daily) and pantoprazole 40 mg twice daily for the duration of one week [20]. Due to the high metronidazole resistance rate in Malaysia [21], [22], metronidazole is not recommended. In cases where the therapy failed, two consecutive rescue regimens are used [23], [24]. The first line rescue regimen consists of rabeprazole 20 mg three times daily and amoxicillin 1 g three times daily for two weeks [23]. Rabeprazole is less susceptible to the influence of genetic polymorphisms for CYP2C19. Thus, it has greater and faster acid suppression compared to older PPIs [25]. Amoxicillin is one of the effective antibiotics to *H. pylori* with few side effects. The antibiotic resistance to amoxicillin has not been reported in Malaysia [26]. Patients who continue to have *H. pylori* infection will be put on the second line rescue regimen which consists of a rabeperazole 20 mg twice daily, levofloxacin 500 mg twice daily and amoxicillin 1 g twice daily for a further 2 weeks [23].

As different regimens were used for treatment, it is crucial to monitor the development of resistance for clarithromycin, amoxicillin, metronidazole and fluoroquinolones periodically to ensure the current therapy regimens remain effective. The assessment on resistance rate to other antibiotics used as rescue therapy in *H. pylori* eradication (such as tetracycline and rifampicin), can also provide a baseline antibiotic resistance profile of locally isolated *H. pylori* strains.

Recent reports suggest that the underlying antibiotic resistance mechanisms are mainly due to the genetic plasticity of *H. pylori* that results in genetic mutations. These include point mutation involving transition of adenine to guanine on position 2142 and 2143 in the domain V of *23S rRNA* gene, which confers resistance towards clarithromycin [27]. Similarly, mutation in gene *rdxA* and *frxA* is reported to confer metronidazole resistance in *H. pylori* [28]. Unlike mutational patterns in *23S rRNA* and *gyrA*, the impact of mutation in gene *rdxA* and *frxA* is caused by the inactivation of the gene function via frameshift mutation, insertions and deletions. On the other hand, mutational changes in quinolone-resistant determination region (QRDR) of *gyrA* gene greatly reduce the eradication efficacy of fluoroquinolones. Almost 90% of the fluoroquinolones-resistant strains harbour mutation at position 87 and 91 of the translational protein sequence gyrase subunit A (*gyrA*) [29]. Mutations on gene *gyrB* known to confer fluoroquinolones resistance have also been reported [17]. Despite that, double mutation in *gyrA* is reported to have a greater impact on fluoroquinolones resistance [30].

In this study, surveillance of conventional and potential antibiotics - comprising of 10 antibiotics, comprising of metronidazole, clarithromycin, fluoroquinolone (ciprofloxacin, moxifloxacin, levofloxacin, and gemifloxacin), amoxicillin, rifampicin, tetracycline, and nitrofurantoin - were included to provide a comprehensive insight of the current *H. pylori* antibiotic resistance prevalence in Malaysia. In addition, correlation between the antibiotic susceptibility patterns and the presence of associated genetic mutations in the local population will also be evaluated.

## Materials and Methods

### Bacterial strains

This study was approved by the University of Malaya Medical Centre (UMMC) Medical Ethics Committee and biopsy samples for culturing were obtained with informed and written consent from consecutive and non-repetitive patients who presented for endoscopy at UMMC. Gastric biopsy samples obtained from antrum and body of the stomach were homogenized in different tubes and plated directly onto non-selective and selective chocolate agar supplemented with 7% lysed horse blood (Oxoid, UK). Selective chocolate agar contained vancomycin (10 µg/ml), amphotericin B (5 µg/ml), trimethoprim (5 µg/ml) and nalidixic acid (20 µg/ml) [31]. The inoculated agar plates were incubated for 3–10 days in a humidified 10% CO_2_ incubator at 37°C. *H. pylori* were successfully cultured from 110 patients and were confirmed by positivity for urease test. Amongst these, 102 were naive to standard *H. pylori* therapy whilst the remaining eight were from patients with treatment failure. Clonal strains derived from well-isolated *H. pylori* colonies of the same patient were tested for homogeneity by random amplification of polymorphic DNA (RAPD) and representative clonal strains were used for this study.

### Antibiotic Susceptibility Test

Bacterial antibiotic susceptibility against a panel of 10 antibiotics, comprising of metronidazole, clarithromycin, fluoroquinolone (ciprofloxacin, moxifloxacin, levofloxacin, and gemifloxacin), amoxicillin, rifampicin, tetracycline, and nitrofurantoin, were tested using Etest strips (Biomerieux, France). In this experiment, *H. pylori* inoculum was prepared from 72 h-old culture and spread confluently on a non-selective chocolate agar plate prior to the placement of the Etest strip. Minimal inhibition concentration (MIC) was measured after three days. The cut-off values adopted were as follow: ≥8.0 µg/ml for metronidazole [32]; ≥1.0 µg/ml for clarithromycin [33]; ≥1.0 µg/ml for fluoroquinolone [29]; ≥2.0 µg/ml, ≥4.0 µg/ml, and ≥1.0 µg/ml for amoxicillin, rifampicin and tetracycline respectively [34]; and ≥4.0 µg/ml for nitrofurantoin [26] were taken as resistant to the particular antibiotic.

### Molecular Detection on Resistant Strains

Mutations in *gyrA, gyrB, rdxA, frxA, 23S rRNA* were assessed on antibiotic-resistant strains by targeted gene sequencing approach. Bacterial genome DNA was extracted using RTP Bacteria DNA Mini Kit (Invitek, Germany). Amplification of 582-bp and 465-bp region on *gyrA* and *gyrB* respectively were performed on fluoroquinolone-resistant strain with thermocycler conditions being described by Wang *et al* [29]. Regions of 425-bp within bacterial 23S rRNA peptidyl transferase gene were amplified from clarithromycin-resistant strains as used by Ho *et al* [27]. For metronidazole-resistant strains, *rdxA* and *frxA* were amplified as described by Han *el al* [32]. The primer sequences are shown in Table 1. PCR products were purified using SV Gel and PCR Clean-Up Kits (Promega, USA) and sequenced on the Sanger method. Comparative sequence analysis between resistant and sensitive strains was carried out using BioEdit and MEGA software [35], [36]. The DNA sequence results were aligned and coordinated to *H. pylori* 26695 (Accession: NC_000915.1; GI: 15644634).

**Table 1:** Oligonucleotide primers for amplifying *rdxA*, *frxA 23S rRNA*, *gyrA* and *gyrB*.

Gene	Primer	Sequence
*rdxA*	rdxAF	5′-ATGGTAATTGTTTCGTTAGGG-3′
	*rdxA*R	5′-CTCCTTGAACTTTAATTTAG-3′
*frxA*	frxAF	5′-TGGATATGGCAGCCGTTTA-3′
	frxAR	5′-GGTTATCAAAAAGCTAACAGCG-3′
*gyrA*	gyrAF	5′-AGCTTATTCCATGAGCGTGA-3′
	gyrAR	5′-TCAGGCCCTTTGACAA ATTC-3′
*gyrB*	gyrBF	5′-CCCTAACGAAGCCAAAATCA-3′
	gyrBR	5′-GGGCGCAAATAACG ATAGAA-3′
*23S rRNA*	HP23F	5′-CCACAGCGATGTGGTCTCAG-3′
	HP23R	5′-CTCCATAAGA GCCAAAGCCC-3′

### Statistical Analysis

The two-tailed Student's *t*-test with unequal variance function of Microsoft Excel was used to determine the statistical significance of differences between unpaired samples. The minimum level of significance was set at <0.05.

## Results

### Prevalence of Antibiotic Resistance

DNA fingerprinting using RAPD demonstrated that of the 102 *H. pylori* strains isolated from each treatment-naive patient, there was no heterogeneous infection involving more than one genotype of *H. pylori* occurring in these patients. Among the primary resistance patterns, metronidazole resistance was the highest (33 strains or 32.3%), followed by clarithromycin (7 strains or 6.8%) and fluoroquinolone (7 strains or 6.8%) (Table 2). Among the fluoroquinolones resistance patterns, all fluoroquinolone-resistant strains were resistant to levofloxacin, ciprofloxacin and moxifloxacin but only four strains (57.1%) were resistant to gemifloxacin. The prevalence of primary multi-drug resistance, where resistance was found towards more than one antimicrobial agent, was demonstrated in five strains (4.9%).

**Table 2:** Primary and secondary antibiotic resistance pattern.

Antibiotics	MIC Range	Resistance strains	
		Primary	Secondary
		n = 102 (%)	n = 8 (%)
Metronidazole	≤0.016–≥256	33 (32.3)	4 (50.0%)
Clarithromycin	≤0.016–64	7 (6.8)	7 (87.5%)
Fluoroquinolone			
Ciprofloxacin	0.064–≥32	7 (6.8)	2 (25.0%)
Levofloxacin	≤0.020–≥32	7 (6.8)	2 (25.0%)
Moxifloxacin	≤0.002–≥32	7 (6.8)	2 (25.0%)
Gemifloxacin	≤0.002–≥32	5 (4.9)	2 (25.0%)
Amoxicillin	≤0.016–0.32	0	0
Rifampicin	0.032–2	0	0
Tetracycline	≤0.016–0.125	0	0
Nitrofurantoin	≤0.032–0.19	0	0

Similarly, among the eight patients with treatment failure, no heterogeneous infection by more than one strain was found based on RAPD analysis. The MIC results revealed that this population of strains consisted of three single-drug resistant (SDR) strains and five multi-drug resistant (MDR) strains (62.5%) (Table 2). Resistance towards amoxicillin, rifampicin, tetracycline, and nitrofurantoin was not detected amongst any of these strains.

### Multi-drug Resistance

Among the MDR strains (n = 10), 90% were resistant to clarithromycin, 70% to metronidazole and 50% to fluoroquinolones. Among these, 5/10 (50%) strains were resistant to both metronidazole and clarithromycin followed by 3/10 (30%) strains resistant to both clarithromycin and fluoroquinolone. One of these ten strains (10%) was resistant to all three antibiotics. Only 1/10 (10%) strain was resistant to both metronidazole and fluoroquinolone. The average MIC of MDR were observed to be higher that SDR for all three antibiotics (Table 3). The two-tailed Student's *t*-test with unequal variance showed that the differences was statistically significant (*P<0.05*) for metronidazole and clarithromycin and highly significant for fluoroquinolone (*P<0.001*).

**Table 3:** Comparison of average minimum inhibitory concentration (MIC) between single-drug resistant (SDR) and MDR strains.

	Metronidazole		Clarithromycin		Fluoroquinolone	
	SDR	MDR	SDR	MDR	SDR	MDR
***N***	30	7	5	3	4	5
**Ave MIC**	152.0	67.1	9.0	24.2	10.8	26.
***p-*** **value**	<0.05		<0.05		<0.001	

Two-tailed Student's *t*-test *P*-value < 0.05 was considered significant and <0.001 was highly significant.

Note: MDR (Multi-drug resistance), SDR (Single-drug resistance).

Ave MIC (Average MIC).

### Genetic Variations of *rdxA* and *frxA* in Metronidazole-resistant Strains

Based on *rdxA* and *frxA* gene variations and amino acid sequences generated among metronidazole-resistant strains, variations of *rdxA* with its corresponding amino acid alteration in RdxA were identified in 26/37 strains (70.2%) (Figure S1). Parallel with this, out of these 37 strains, 21 (56.8%) demonstrated the presence of truncated RdxA. The cause of premature termination was due to introduction of a stop codon by single amino acid substitution and frame-shift alteration. Although the other 16 strains encoded for full length RdxA, 3 (8.1%) were found to have nucleotides insertion/deletion resulting in amino acid insertion and deletion and frame-shift mutation at the C-terminal (Figure S2). In addition, one strain (2.7%) was found to have the stop codon at the end of *rdxA* missing. Similarly, variation of *frxA* and alteration in FrxA were identified in 25/37 (67.6%) metronidazole-resistant strains (Figure S3). Among these strains premature termination codon (PTC) was present in 20 strains (54.1%) and five out of 17 (13.5%) strains had single or double amino acid deletion or insertion at the *frxA* (Figure S4).

Taken together, 18/37 (48.6%) of the metronidazole-resistant strains isolated had mutations in both *rdxA and frxA*; 8/37 (21.6%) in *rdxA* only; 7/37 (18.9%) in *frxA* only; 4/37 (10.8%) had no mutation in both genes (Table 4). No apparent correlation was identified between degree of metronidazole resistance with type and number of mutations in *rdxA* and *frxA*.

**Table 4:** MIC of metronidazole and *rdxA* and *frxA* mutations.

	Type of		Mutations		At lease one
Strains	resistance	MIC(µg/ml)	*rdxA*	*frxA*	mutation
**UM003**	Primary	>256	PTC	N*	Yes
**UM005**	Primary	16	PTC	148delM	Yes
**UM008**	Primary	>256	N*	N*	No
**UM010**	Primary	12	N*	N*	No
**UM019**	Primary	12	N*	PTC	Yes
**UM023**	Primary	>256	PTC	N*	Yes
**UM034**	Primary	>256	PTC	PTC	Yes
**UM045**	Primary	64	PTC	PTC	Yes
**UM051**	Primary	>256	PTC	N*	Yes
**UM054**	Primary	12	N*	N*	No
**UM067**	Primary	16	PTC	PTC	Yes
**UM074**	Primary	>256	PTC	PTC	Yes
**UM080**	Primary	24	82_84insALM	N*	Yes
**UM084**	Primary	12	38_40delEIA	PTC	Yes
**UM087(MDR)**	Primary	6	N*	PTC	Yes
**UM090**	Primary	48	PTC	PTC	Yes
**UM094**	Primary	>256	PTC	207delW	Yes
**UM096**	Secondary	>256	PTC	207delW	Yes
**UM106**	Primary	8	N*	106insG	Yes
**UM111(MDR)**	Secondary	32	N*	PTC	Yes
**UM114(MDR)**	Primary	64	N*	PTC	Yes
**UM118**	Primary	>256	PTC	N*	Yes
**UM136**	Primary	>256	PTC	PTC	Yes
**UM139(MDR)**	Primary	>256	N*	PTC	Yes
**UM144**	Primary	64	N*	N*	No
**UM146**	Primary	256	PTC	PTC	Yes
**UM148**	Primary	>256	stop211V	PTC	Yes
**UM157(MDR)**	Primary	24	PTC	N*	Yes
**UM158(MDR)**	Secondary	64	PTC	PTC	Yes
**UM196(MDR)**	Secondary	24	PTC	N*	Yes
**UM209**	Primary	>256	N*	PTC	Yes
**UM215**	Primary	>256	PTC	PTC	Yes
**UM237**	Primary	>256	PTC	N*	Yes
**C010**	Primary	64	PTC	PTC	Yes
**C018**	Primary	256	196frameshift	PTC	Yes
**C020**	Primary	16	72insF	PTC	Yes
**C039**	Primary	96	PTC	165_166delGY	Yes

Note: MDR (Multi-drug resistant), PTC (Premature termination codon).

N*: no specific variation,

ins: amino acid insertion, del: amino acid deletion.

### Genetic Variation at V domain of *23S rRNA* in Clarithromycin-resistant Strains

V domain of *23SrRNA* (nucleotide 1876-2201) sequenced in 14 clarithromycin-resistant strains demonstrated this region to be highly conserved with minimal nucleotide variation in comparison to reference genome *H. pylori* 26695 (HP0949) (Table 5) (Figure S5). Two interesting point mutations, which include the single nucleotide transition at A2142G (1/14 or 7.1%) and A2143G (12/14 or 85.7%), were observed. None of these strains were found to have double mutations of A2142G and A2143G but either of these mutations occurred in 13/14 (92.8%) resistant strains. None of these mutations were found among the susceptible strains. The strain harbouring no mutation at 2142 or 2143 was found to have only marginal level of resistance to clarithromycin. Overall, there was no correlation between levels of MIC for clarithromycin and the sites of mutation.

**Table 5:** MIC of clarithromycin and *23S rRNA* mutations.

Strains	Type of resistance	MIC (µg/ml)	Mutations on *23S rRNA*
**UM037**	Primar	16	A2142G
**UM038(MDR)**	Primary	32	A2143G, T2182C
**UM085(MDR)**	Secondary	16	A2143G, T2182C
**UM087(MDR)**	Primary	6	A2143G, T2182C
**UM097(MDR)**	Secondary	32	A2143G, T2182C
**UM111(MDR)**	Secondary	32	A2143G
**UM119**	Secondary	2	N*
**UM139(MDR)**	Primary	64	A2143G
**UM147**	Primary	12	A2143G, T2182C
**UM157(MDR)**	Primary	8	A2143G
**UM158(MDR)**	Secondary	4	A2143G, T2182C
**UM196(MDR)**	Secondary	24	A2143G, T2182C
**UM229**	Primary	3	A2143G, T2182C
**C021**	Secondary	12	A2143G, T2182C

Note: MDR (Multi-drug resistance).

N*: no specific variation.

### Amino Acid Variation at QRDR region of *gyrA* and *gyrB* in Fluoroquinolone-resistant Strains

Selective regions of the Quinolone Resistance Determining Region (QRDR) of *gyrA* (nucleotide 142-606) and *gyrB* (nucleotide 1180-1491) were sequenced from all nine resistant strains and five susceptible strains. Upon putative translation, three different amino acid variants at gyrase A subunit were detected to be exclusively present in these resistant strains only. Five out of nine resistant strains exhibited amino acid substitution at position Asp-91 (55.6%), whereas substitution at site Asn-87 was present in four other strains (44.4%). Besides that, 4/9 resistant strains presented with substitution at Val-199 (44.4%) of which three were in association with Asp-91 and one with Asn-87 (Table 6) (Figure S6).

**Table 6:** MIC of fluoroquinolone and *gyrA* and *gyrB* mutations.

Strains	Type of	MIC (µg/ml)				Mutations	
	resistance	CIP	LEV	MOX	GEM	*gyrA*	*gyrB*
**UM038(MDR)**	Primary	24	>32	>32	16	D91Y	N*
**UM077**	Primary	4	1.5	>32	0.5	N87K	N*
**UM085(MDR)**	Secondary	>32	>32	>32	2	N87K	N*
**UM087(MDR)**	Primary	>32	>32	>32	>32	D91G, V199I	D481E
**UM097(MDR)**	Secondary	32	32	>32	3	N87K	F438S
**UM114(MDR)**	Primary	4	>32	>32	>32	N87K, V199A	D481E, R484K
**UM122**	Primary	>32	>32	>32	4	D91G, V199I	N*
**UM165**	Primary	6	6	6	0.5	D91N, V199I	D484K
**UM131**	Primary	4	4	8	0.5	D91N	D481E, D484K

Note: MDR (Multi-drug resistance).

N*: no specific variation.

On the other hand, two amino acid variants at region of study of gyrase subunit B were observed exclusively in fluoroquinolone resistant strains. Three out of nine strains presented with amino acid substitutions at each position Asp-481 (33.3%) and Arg-484 (33.3%) (Table 6). One strain with high MIC presented with mutation on *gyrB* at Phe-438 into Serine and co-occured with mutation at Asn-87 at *gyrA*. Four out of nine fluoroquinolone-resistant strains did not contain any mutations in *gyrB* (44.4%). There was no apparent correlation between degree of fluoroquinolone resistance with type and number of mutations in *gyrA* and *gyrB*.

## Discussion

Generally, in Malaysia, the prevalence of metronidazole-resistant *H. pylori* strains is high [21]. This sustained high level of metronidazole resistance prior to *H. pylori* eradication therapy could be attributed to massive prescription of metronidazole as a common anti-parasite drug and for other gynaecologic diseases in many developing countries [37]. Moreover, these high prevalence of metronidazole resistance rate has also been well-documented in neighbouring Southeast Asian countries such as Vietnam (69.9%) [38], Thailand (30.4%) [39], and Singapore (31.7%) [40]. Therefore, in agreement with other previous studies [21], [26], we have further confirmed that metronidazole should not be the choice for first line *H. pylori* eradication therapy in the local population [20].

Unlike metronidazole, the resistance rate of clarithromycin is relatively low in Malaysia compared to other neighboring countries such as Vietnam (33%) [38] and Thailand (13.8%) [41]. The underlying reason on why clarithromycin resistance rate in Malaysia remains low is not clear even though increased clarithromycin resistance, especially in European countries, has been reported [42]. Although also indicated for respiratory infections, skin infections and Lyme disease, clarithromycin has not been popular in clinical practice locally due to high cost and side effects. We speculated that this could be the factor that causes relatively low resistance rate been observed in this study [27]. However, clarithromycin resistance rate is definitely on the rise as shown in this study in comparison to previous study [21], [26], which is of growing concern. Clarithromycin is the key antibiotic in the *H. pylori* therapy regimen. Emergence of resistance rate could eventually reduces the efficacy of clarithromycin-based therapy similar to that of western countries as highlighted by Fischbach [10]. Megraud reviewed that resistance to clarithromycin is highly correlated to use of these drugs for non-*H. pylori* infectious diseases [11]. Therefore, even though the emergence of clarithromycin-resistant strains in Malaysia is progressively slower than other countries, strict guidelines on the use of clarithromycin should be made to maintain a low level of resistance [43].

In the local population, fluoroquinolone appears to be superior in secondary or tertiary rescue regimens and proven to show high eradication rate [23], [24]. In contrast to previous reporting of zero resistance to levofloxacin in Malaysia [26], we found a low but significant increase of fluoroquinolone resistance, which is of concern too. The developing of fluoroquinolone resistance is rapid and closely dependent on increasing usage of this class of antibiotic [11]. Besides, a cross resistance pattern was observed across different generations of fluoroquinolone (moxifloxacin > levofloxacin > ciprofloxacin > gemifloxacin) with high chance of developing fluoroquinolone resistance as a result of other treatments prior to *H. pylori* eradication. Similar to an early report on the Taiwanese population [15], the degree of resistance to gemifloxacin was low in the Malaysian population suggested that it may have better drug efficacy than levofloxacin in *H. pylori* eradication in our population.

Multi-drug resistance is highly prevalent among secondary resistant strains suggested that MDR strains were likely the result of selective pressure due to previous antibiotic exposure from either *H. pylori* treatment or treatment of unrelated infections. Interestingly, since most MDR strains were clarithromycin-resistant, data from this study further suggested that there may be an underlying mechanism influencing the selection and evolution of MDR strains, which is more prevalently found among clarithromycin-resistant strains. Furthermore, this common underlying mechanism of MDR may directly or indirectly have led to a higher level of resistance, which may explain why high MICs were more commonly associated with MDR than SDR strains. However, these deductions about MDR strains are not conclusive given the small number of MDR strains available in this study and further study is necessary. Nevertheless, it further emphasised the importance to keep clarithromycin resistance under close surveillance.

The correlation between *rdxA* and *frxA* mutations and metronidazole resistance in *H. pylori* has been well-demonstrated [28]. Many point mutations have been shown to exist in *rdxA* and *frxA* but these positions were not uniform across all geographical regions [44]. Therefore, *rdxA* and *frxA* mutations were analyzed by comparing between local metronidazole-sensitive and metronidazole-resistant strains. As expected, this study demonstrated that nucleotide alterations in *rdxA* and *frxA* in resistant strains were random and highly distributed. Accumulatively, 89.1% of metronidazole-resistant strains possessed PTC or insertion/deletion at one or both *rdxA* and *frxA*. However, 10.8% of metronidazole-resistant strains did not contain any alteration in both *rdxA* and *frxA*. In this small subset of metronidazole-resistant strains, mutations may be present on other redox enzymes. However, screening for mutations in *rdxA* and *frxA* should be sufficient to identify about 89% of metronidazole-resistant strains in the local population.

In order to include mutations other than A2142G and A2143G, direct DNA sequencing of the amplicon was applied in this study instead of using *Bbs*I and *Bsa*I restriction method as described in previous study [27]. There was a conflict in stating either A2142G or A2143G mutation as predominant in clarithromycin-resistant strains. Our data agreed with investigators who study on Asian strains whereby the A2143G mutation was prominent. Therefore, this further confirms that the predominant of mutation sites could be strain-specific and differs between Western and Asian strains [45], [46]. Apart from that, some investigators proposed that T2183C mutation may play a role in clarithromycin resistance [47]. Contradictory to that, T2183C mutation was detected in both resistant (9 out of 14 or 64.2%) and sensitive (5 out of 8 or 62.5%) strains. Furthermore, dual mutation on A2142G or A2143G and T2183C did not result in higher MIC. Thus, we concluded that T2183C mutation was not directly responsible or synergistically involved in conferring clarithromycin resistance. Screening for A2142G or A2143G mutations of the *23S rRNA* was able to detect 92.9% of clarithromycin-resistant strains in the Malaysian population.

Consistent with other studies [48], [49], [50], sequencing on *gyrA* revealed the exclusive correlation of Asn-87 and Asp-91 with fluoroquinolone resistance. Apart from that, substitution at codon Val-199 was also observed in our strains. However, the correlation of dual mutation of Asn-87 or Asp-91 and Val-199 with level of fluoroquinolone resistance was poor. The only explanation is that the amino acid substitution on Val-199 did not have much impact to the structure of gyrase subunit A since the substitution from valine to isoleucine or alanine does not change the side-chain polarity. On the other hand, mutational changes on *gyrB* did not show high correlation with fluoroquinolone resistance too. Our data agreed with previous study by Tankovic *et al* that demonstrated some amino acid substitution is also present in sensitive strains [30]. In agreement with other studies on newer fluoroquinolone drugs, our data shows that gemifloxacin was active against some but not all strains displaying resistance to ciprofloxacin, levofloxacin and moxifloxacin [15], [51]. The phenomenon did not correlate with any mutational patterns detected in our study indicating possibility of other mechanism that act synergistically in fluoroquinolone resistance. The screening for Asn-87 and Asp-91 mutations on *gyrA* is recommended for our local population.

In summary, this study shows that resistance rate of metronidazole remained high. Despite the current low prevalence of clarithromycin and fluoroquinolone resistance, in the Malaysian population, the trend is on the rise and situation warrants close monitoring.

## Supporting Information

Figure S1 **Premature truncation in peptide translation of** ***rdxA*** **.**
(DOCX)

Figure S2 **Alignment of peptide sequence with reference and sensitive strains for** ***rdxA*** **.**
(DOCX)

Figure S3 **Premature truncation in peptide translation of** ***frxA*** **.**
(DOCX)

Figure S4 **Alignment of peptide sequence with reference and sensitive strains for** ***frxA*** **.**
(DOCX)

Figure S5 **Alignment of nucleotides sequence with reference and sensitive strains for** ***23S rRNA*** **.**
(DOCX)

Figure S6 **Alignment of peptide sequence with reference and sensitive strains for** ***gyrA*** **and** ***gyrB*** **.**
(DOCX)

## References

[1] Dunn BE, Cohen H, Blaser MJ (1997) *Helicobacter pylori* . Clin Microbiol Rev 10: 720–741. 9336670 10.1128/cmr.10.4.720 PMC172942

[2] Dorer MS, Talarico S, Salama NR (2009) *Helicobacter pylori*'s unconventional role in health and disease. PLoS Pathog 5: e1000544. 19855816 10.1371/journal.ppat.1000544 PMC2729406

[3] WHO (2011) Agents Classified by the IARC Monographs, Volumes 1–102. Cancer, World Health Organization.

[4] Goh KL, Chan WK, Shiota S, Yamaoka Y (2011) Epidemiology of *Helicobacter pylori* infection and public health implications. Helicobacter 16 Suppl 1 1–9. 10.1111/j.1523-5378.2011.00874.x PMC3719046 21896079

[5] Tan HJ, Goh KL (2008) Changing epidemiology of *Helicobacter pylori* in Asia. Journal of Digestive Diseases 9: 186–189. 18959588 10.1111/j.1751-2980.2008.00344.x

[6] Wu CY, Kuo KN, Wu MS, Chen YJ, Wang CB, et al (2009) Early *Helicobacter pylori* Eradication Decreases Risk of Gastric Cancer in Patients With Peptic Ulcer Disease. Gastroenterology 137: 1641–1648. 19664631 10.1053/j.gastro.2009.07.060

[7] Malfertheiner P, Megraud F, O'Morain C, Bell D, Bianchi Porro G, et al (1997) Current European concepts in the management of *Helicobacter pylori* infection—the Maastricht Consensus Report. The European *Helicobacter pylori* Study Group (EHPSG). Eur J Gastroenterol Hepatol 9: 1–2. 9031888 10.1097/00042737-199701000-00002

[8] Malfertheiner P, Megraud F, O'Morain CA, Atherton J, Axon ATR, et al (2012) Management of *Helicobacter pylori* infection-the Maastricht IV/Florence Consensus Report. Gut 61: 646–664. 22491499 10.1136/gutjnl-2012-302084

[9] Zullo A, De Francesco V, Vaira D (2011) Sequential therapy for *Helicobacter pylori* eradication: is levofloxacin better? Gut 60: 1604–1604. 21193446 10.1136/gut.2010.231233

[10] Fischbach L, Evans EL (2007) Meta-analysis: the effect of antibiotic resistance status on the efficacy of triple and quadruple first-line therapies for *Helicobacter pylori* . Alimentary Pharmacology & Therapeutics 26: 343–357. 17635369 10.1111/j.1365-2036.2007.03386.x

[11] Megraud F (2012) The challenge of *Helicobacter pylori* resistance to antibiotics: the comeback of bismuth-based quadruple therapy. Therap Adv Gastroenterol 5: 103–109. 10.1177/1756283X11432492 PMC3296089 22423259

[12] Gisbert JP (2008) Second-line rescue therapy with levofloxacin after H-pylori treatment failure: A Spanish multicenter study. Gastroenterology 134: A333–A333.

[13] Katelaris PH, Forbes GM, Talley NJ, Crotty B (2002) A randomized comparison of quadruple and triple therapies for *Helicobacter pylori* eradication: The QUADRATE Study. Gastroenterology 123: 1763–1769. 12454831 10.1053/gast.2002.37051

[14] Cuadrado-Lavin A, Salcines-Caviedes JR, Carrascosa MF, Mellado P, Monteagudo I, et al (2012) Antimicrobial susceptibility of *Helicobacter pylori* to six antibiotics currently used in Spain. J Antimicrob Chemother 67: 170–173. 21965436 10.1093/jac/dkr410

[15] Chang WL, Kao CY, Wu CT, Huang AH, Wu JJ, et al (2012) Gemifloxacin Can Partially Overcome Quinolone Resistance of *H. pylori* with *gyrA* Mutation in Taiwan. Helicobacter 17: 210–215. 22515359 10.1111/j.1523-5378.2012.00935.x

[16] Kim N, Lee JW, Kim JN, Lee DH, Jung HC, et al (2011) Mutations of *Helicobacter pylori* in Fluoroquinolone Resistance and the Effect of Point Mutation of *gyrA* on the Second Line Eradication Therapy in Korea. Gastroenterology 140: S881–S881.

[17] Rimbara E, Noguchi N, Kawai T, Sasatsu M (2012) Fluoroquinolone Resistance in *Helicobacter pylori*: Role of Mutations at Position 87 and 91 of GyrA on the Level of Resistance and Identification of a Resistance Conferring Mutation in GyrB. Helicobacter 17: 36–42. 10.1111/j.1523-5378.2011.00912.x 22221614

[18] Bago J, Majstorovic K, Belosic-Halle Z, Kucisec N, Bakula V, et al (2010) Antimicrobial resistance of *H. pylori* to the outcome of 10-days vs. 7-days Moxifloxacin based therapy for the eradication: a randomized controlled trial. Ann Clin Microbiol Antimicrob 9: 13. 20398300 10.1186/1476-0711-9-13 PMC2867970

[19] Gao W, Cheng H, Hu F, Li J, Wang L, et al (2010) The evolution of *Helicobacter pylori* antibiotics resistance over 10 years in Beijing, China. Helicobacter 15: 460–466. 21083752 10.1111/j.1523-5378.2010.00788.x

[20] Qua CS, Manikam J, Goh KL (2010) Efficacy of 1-week proton pump inhibitor triple therapy as first-line *Helicobacter pylori* eradication regime in Asian patients: is it still effective 10 years on? J Dig Dis 11: 244–248. 20649738 10.1111/j.1751-2980.2010.00445.x

[21] Ahmad N, Zakaria WR, Mohamed R (2011) Analysis of antibiotic susceptibility patterns of *Helicobacter pylori* isolates from Malaysia. Helicobacter 16: 47–51. 21241412 10.1111/j.1523-5378.2010.00816.x

[22] Ahmad N, Zakaria WR, Abdullah SA, Mohamed R (2009) Characterization of clarithromycin resistance in Malaysian isolates of *Helicobacter pylori* . World Journal of Gastroenterology 15: 3161–3165. 19575497 10.3748/wjg.15.3161 PMC2705740

[23] Goh KL, Manikam J, Qua CS (2012) High-dose rabeprazole–amoxicillin dual therapy and rabeprazole triple therapy with amoxicillin and levofloxacin for 2 weeks as first and second line rescue therapies for *Helicobacter pylori* treatment failures. Alimentary Pharmacology & Therapeutics 35: 1097–1102. 22404486 10.1111/j.1365-2036.2012.05054.x

[24] Goh KL (2012) Letter: third-line rescue therapy with levofloxacin after failure of two treatments to eradicate *Helicobacter pylori* infection - author's reply. Alimentary Pharmacology & Therapeutics 35: 1486–1486. 10.1111/j.1365-2036.2012.05117.x 22582841

[25] Baldwin CM, Keam SJ (2009) Rabeprazole A Review of its Use in the Management of Gastric Acid-Related Diseases in Adults. Drugs 69: 1373–1401. 19583455 10.2165/00003495-200969100-00007

[26] Goh KL, Navaratnam P (2011) High *Helicobacter pylori* resistance to metronidazole but zero or low resistance to clarithromycin, levofloxacin, and other antibiotics in Malaysia. Helicobacter 16: 241–245. 21585611 10.1111/j.1523-5378.2011.00841.x

[27] Ho SL, Tan EL, Sam CK, Goh KL (2010) Clarithromycin resistance and point mutations in the *23S rRNA* gene in *Helicobacter pylori* isolates from Malaysia. Journal of Digestive Diseases 11: 101–105. 20402836 10.1111/j.1751-2980.2010.00423.x

[28] Gerrits MM, van der Wouden EJ, Bax DA, van Zwet AA, van Vliet AH, et al (2004) Role of the *rdxA* and *frxA* genes in oxygen-dependent metronidazole resistance of *Helicobacter pylori* . J Med Microbiol 53: 1123–1128. 15496391 10.1099/jmm.0.45701-0

[29] Wang LH, Cheng H, Hu FL, Li J (2010) Distribution of *gyrA* mutations in fluoroquinolone-resistant *Helicobacter pylori* strains. World J Gastroenterol 16: 2272–2277. 20458765 10.3748/wjg.v16.i18.2272 PMC2868221

[30] Tankovic J, Lascols C, Sculo Q, Petit JC, Soussy CJ (2003) Single and double mutations in *gyrA* but not in *gyrB* are associated with low- and high-level fluoroquinolone resistance in *Helicobacter pylori* . Antimicrob Agents Chemother 47: 3942–3944. 14638505 10.1128/AAC.47.12.3942-3944.2003 PMC296230

[31] Corry JEL, Curtis GDW, Baird RM (2003) Handbook of culture media for food microbiology. Amsterdam Boston: Elsevier. xiv, 662 p.p.

[32] Han F, Liu S, Ho B, Yan Z, Yan X (2007) Alterations in *rdxA* and *frxA* genes and their upstream regions in metronidazole-resistant *Helicobacter pylori* isolates. Res Microbiol 158: 38–44. 17113269 10.1016/j.resmic.2006.10.001

[33] Hirata K, Suzuki H, Nishizawa T, Tsugawa H, Muraoka H, et al (2010) Contribution of efflux pumps to clarithromycin resistance in *Helicobacter pylori* . J Gastroenterol Hepatol 25 Suppl 1 S75–79. 20586871 10.1111/j.1440-1746.2009.06220.x

[34] Wuppenhorst N, Lenze F, Ross M, Kist M (2011) Isolation and eradication of a clinical isolate of *Helicobacter pylori* resistant to five antimicrobials in Germany. J Antimicrob Chemother 66: 222–223. 21051371 10.1093/jac/dkq405

[35] Hall TA (1999) BioEdit: a user-friendly biological sequence alignment editor and analysis program for Windows 95/98/NT. Nucleic Acids Symposium: 95–98.

[36] Tamura K, Peterson D, Peterson N, Stecher G, Nei M, et al (2011) MEGA5: molecular evolutionary genetics analysis using maximum likelihood, evolutionary distance, and maximum parsimony methods. Mol Biol Evol 28: 2731–2739. 21546353 10.1093/molbev/msr121 PMC3203626

[37] Farhana F, Jamaiah I, Rohela M, Aldul-Aziz NM, Nissapatorn V (2009) A ten year (1999-2008) retrospective study of amoebiasis in University Malaya Medical Centre (UMMC), Kuala Lumpur, Malaysia. Tropical Biomedicine 26: 262–266. 20237439

[38] Binh TT, Shiota S, Nguyen LT, Ho DD, Hoang HH, et al (2013) The incidence of primary antibiotic resistance of *Helicobacter pylori* in Vietnam. J Clin Gastroenterol 47: 233–238. 23090037 10.1097/MCG.0b013e3182676e2b PMC3556356

[39] Tangmankongworakoon N, Mahachai V, Thong-Ngam D, Vilaichone RK, Tumwasorn S, et al (2003) Pattern of drug resistant *Helicobacter pylori* in dyspeptic patients in Thailand. J Med Assoc Thai 86 Suppl 2 S439–444. 12930022

[40] Lui SY, Yeoh KG, Ho B (2003) Metronidazole-resistant *Helicobacter pylori* is more prevalent in patients with nonulcer dyspepsia than in peptic ulcer patients in a multiethnic Asian population. J Clin Microbiol 41: 5011–5014. 14605132 10.1128/JCM.41.11.5011-5014.2003 PMC262534

[41] Hansomburana P, Anantapanpong S, Sirinthornpunya S, Chuengyong K, Rojborwonwittaya J (2012) Prevalence of single nucleotide mutation in clarithromycin resistant gene of *Helicobacter pylori*: a 32-months prospective study by using hybridization real time polymerase chain reaction. J Med Assoc Thai 95 Suppl 3 S28–35. 22619884

[42] Agudo S, Perez-Perez G, Alarcon T, Lopez-Brea M (2011) Rapid detection of clarithromycin resistant *Helicobacter pylori* strains in Spanish patients by polymerase chain reaction-restriction fragment length polymorphism. Rev Esp Quimioter 24: 32–36. 21412667 PMC4060515

[43] Lee JW, Kim N, Kim JM, Nam RH, Chang H, et al (2013) Prevalence of Primary and Secondary Antimicrobial Resistance of *Helicobacter pylori* in Korea from 2003 through 2012. Helicobacter 18: 206–214. 23241101 10.1111/hel.12031

[44] Paul R, Postius S, Melchers K, Schafer KP (2001) Mutations of the *Helicobacter pylori* genes *rdxA* and *pbp1* cause resistance against metronidazole and amoxicillin. Antimicrobial Agents and Chemotherapy 45: 962–965. 11181392 10.1128/AAC.45.3.962-965.2001 PMC90405

[45] Liu Z, Shen J, Zhang L, Shen L, Li Q, et al (2008) Prevalence of A2143G mutation of *H. pylori*-*23S rRNA* in Chinese subjects with and without clarithromycin use history. BMC Microbiol 8: 81. 18507832 10.1186/1471-2180-8-81 PMC2427034

[46] Lee JH, Shin JH, Roe IH, Sohn SG, Kang GH, et al (2005) Impact of clarithromycin resistance on eradication of *Helicobacter pylori* in infected adults. Antimicrob Agents Chemother 49: 1600–1603. 15793150 10.1128/AAC.49.4.1600-1603.2005 PMC1068646

[47] Khan R, Nahar S, Sultana J, Ahmad MM, Rahman M (2004) T2182C mutation in *23S rRNA* is associated with clarithromycin resistance in *Helicobacter pylori* isolates obtained in Bangladesh. Antimicrob Agents Chemother 48: 3567–3569. 15328128 10.1128/AAC.48.9.3567-3569.2004 PMC514733

[48] Garcia M, Raymond J, Garnier M, Cremniter J, Burucoa C (2012) Distribution of spontaneous *gyrA* mutations in 97 fluoroquinolone-resistant *Helicobacter pylori* isolates collected in France. Antimicrob Agents Chemother 56: 550–551. 22064536 10.1128/AAC.05243-11 PMC3256052

[49] Kese D, Gubina M, Kogoj R, Strasek K, Tepes B (2009) Detection of point mutations in the *gyrA* gene of *Helicobacter pylori* isolates in Slovenia. Hepatogastroenterology 56: 925–929. 19621731

[50] Nishizawa T, Suzuki H, Kurabayashi K, Masaoka T, Muraoka H, et al (2006) Gatifloxacin resistance and mutations in gyra after unsuccessful *Helicobacter pylori* eradication in Japan. Antimicrob Agents Chemother 50: 1538–1540. 16569878 10.1128/AAC.50.4.1538-1540.2006 PMC1426923

[51] Saravolatz LD, Leggett J (2003) Gatifloxacin, gemifloxacin, and moxifloxacin: the role of 3 newer fluoroquinolones. Clin Infect Dis 37: 1210–1215. 14557966 10.1086/378809

