# Supplementary material for: Functional and Molecular Surveillance of Helicobacter pylori Antibiotic Resistance in Kuala Lumpur
Source: PLoS One. 2014 Jul 8;9(7):e101481. doi: 10.1371/journal.pone.0101481 (PMC4086822; doi:10.1371/journal.pone.0101481)
Supplement: Figure S3 — Premature truncation in peptide translation of frxA . (DOCX) [file pone.0101481.s003.docx]

Figure S3

Premature truncation in peptide translation of gene *frxA.*

Note: Asterisk (*) indicates the stop codon.
