# Supplementary material for: Functional and Molecular Surveillance of Helicobacter pylori Antibiotic Resistance in Kuala Lumpur
Source: PLoS One. 2014 Jul 8;9(7):e101481. doi: 10.1371/journal.pone.0101481 (PMC4086822; doi:10.1371/journal.pone.0101481)
Supplement: Figure S4 — Alignment of peptide sequence with reference and sensitive strains for frxA . (DOCX) [file pone.0101481.s004.docx]

Figure S4

Alignment of peptide sequence with reference and sensitive strains for gene *frxA*.

Note: Dot (.) indicates similar amino acids aligned with the reference; gap (-) indicates no amino acid match with that particular alignment. Reference strains: 26695. Metronidazole sensitive strains: UM035, UM038, UM064, UM113, UM124.
