# Supplementary material for: Functional and Molecular Surveillance of Helicobacter pylori Antibiotic Resistance in Kuala Lumpur
Source: PLoS One. 2014 Jul 8;9(7):e101481. doi: 10.1371/journal.pone.0101481 (PMC4086822; doi:10.1371/journal.pone.0101481)
Supplement: Figure S5 — Alignment of nucleotides sequence with reference and sensitive strains for 23S rRNA . (DOCX) [file pone.0101481.s005.docx]

Figure S5

Alignment of nucleotides sequence with reference and sensitive strains for *23S rRNA*

Note: Dot (.) indicates similar nucleotides aligned with the reference; Reference strains: UM802. Clarithromycin sensitive strains: UM035, UM064, UM104, UM113, UM114, UM122, UM124. UM165
